# Supplementary material for: Salivary Lipids of Patients with Non-Small Cell Lung Cancer Show Perturbation with Respect to Plasma
Source: Int J Mol Sci. 2023 Sep 19;24(18):14264. doi: 10.3390/ijms241814264 (PMC10531690; doi:10.3390/ijms241814264)
Supplement: Supplementary file 1 [file ijms-24-14264-s001.zip › ijms-2568710-supplementary.pdf]

Supplementary Material for

Salivary Lipids of Patients with Non-Small Cell Lung Cancer Show Perturbation with Respect to Plasma

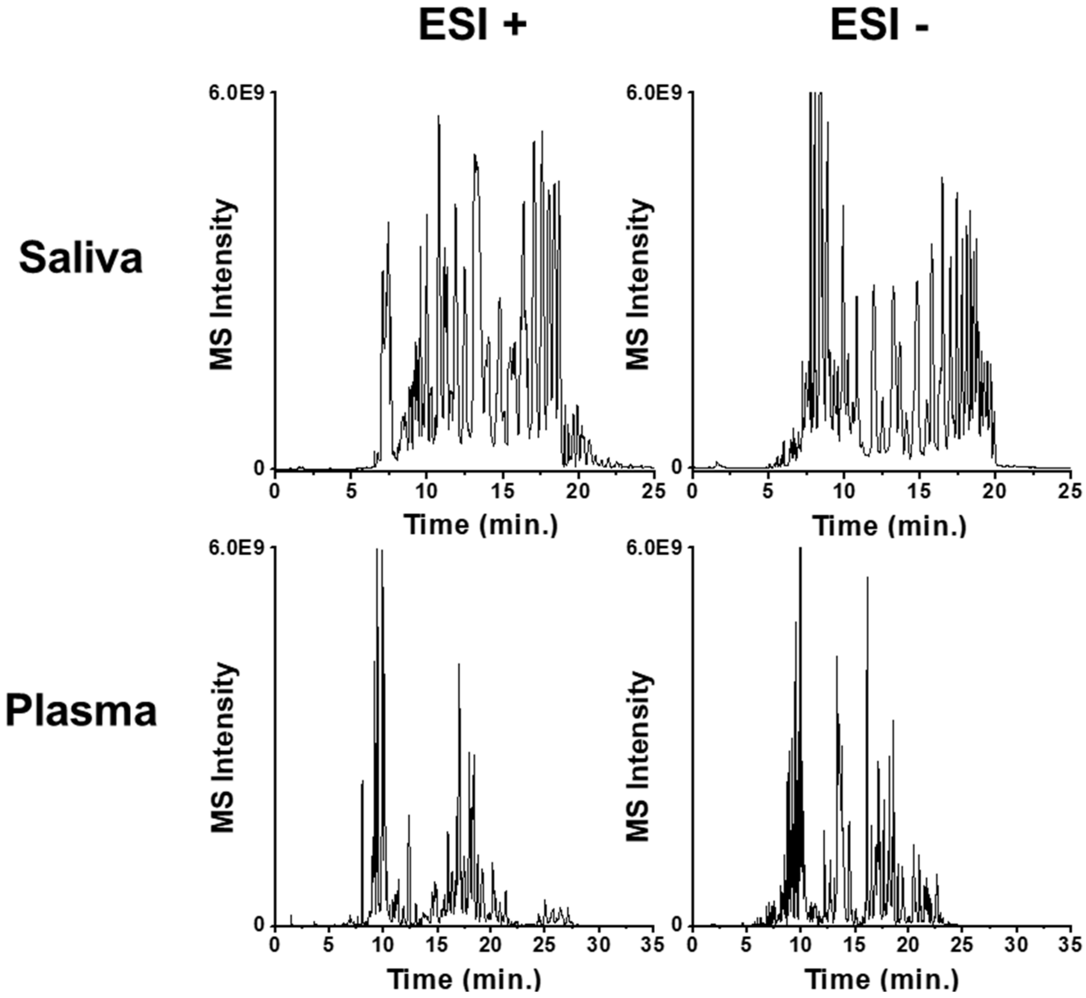

Figure S1. Base peak chromatograms of lipid extracts from saliva and plasma of patients with NSCLC at positive (ESI+) and negative (ESI-) ion modes of nUHPLC-ESI-MS/MS.

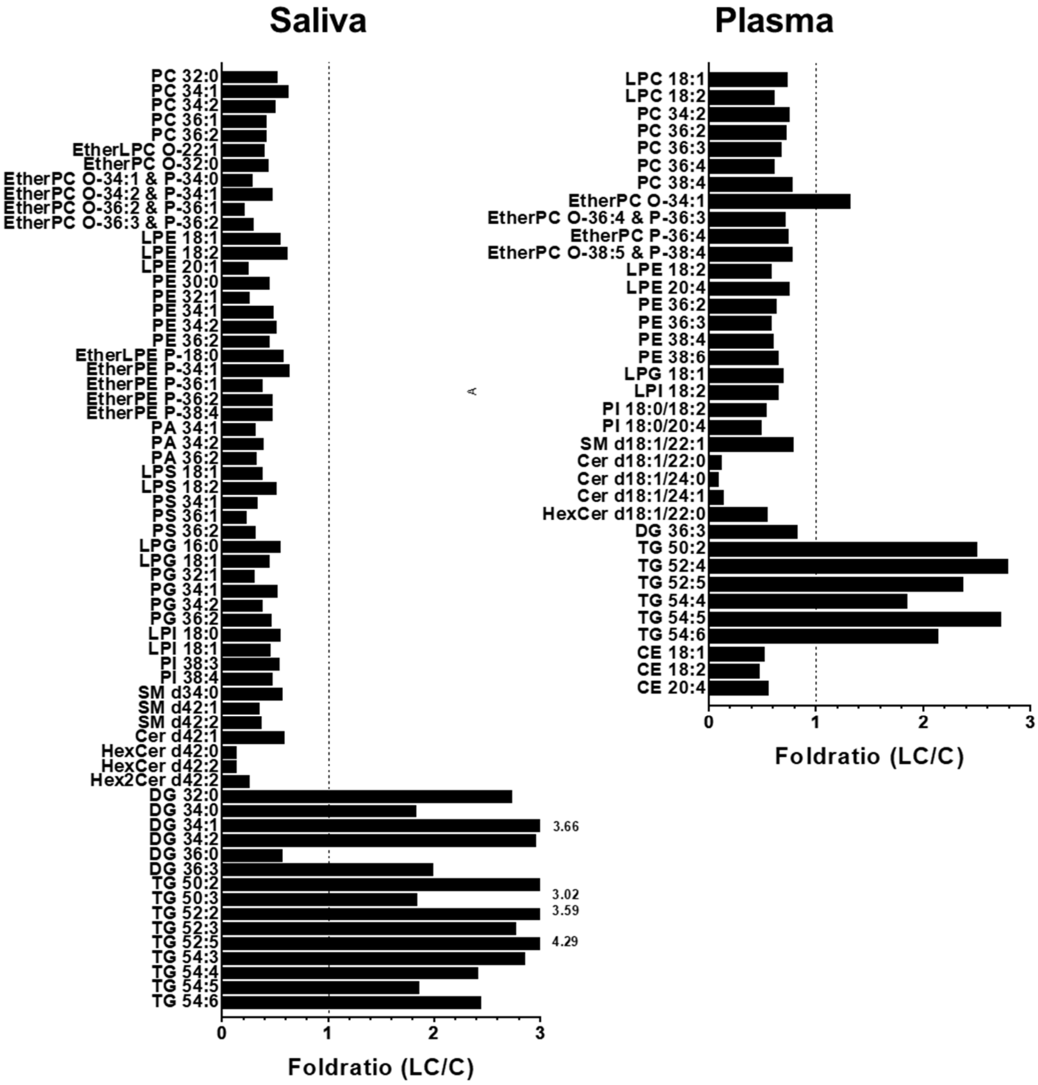

Figure S2. Bar graphs showing fold ratio (LC/C) of each lipid species with statistical significance (p < 0.05) in saliva and plasma samples with LC compared to controls.

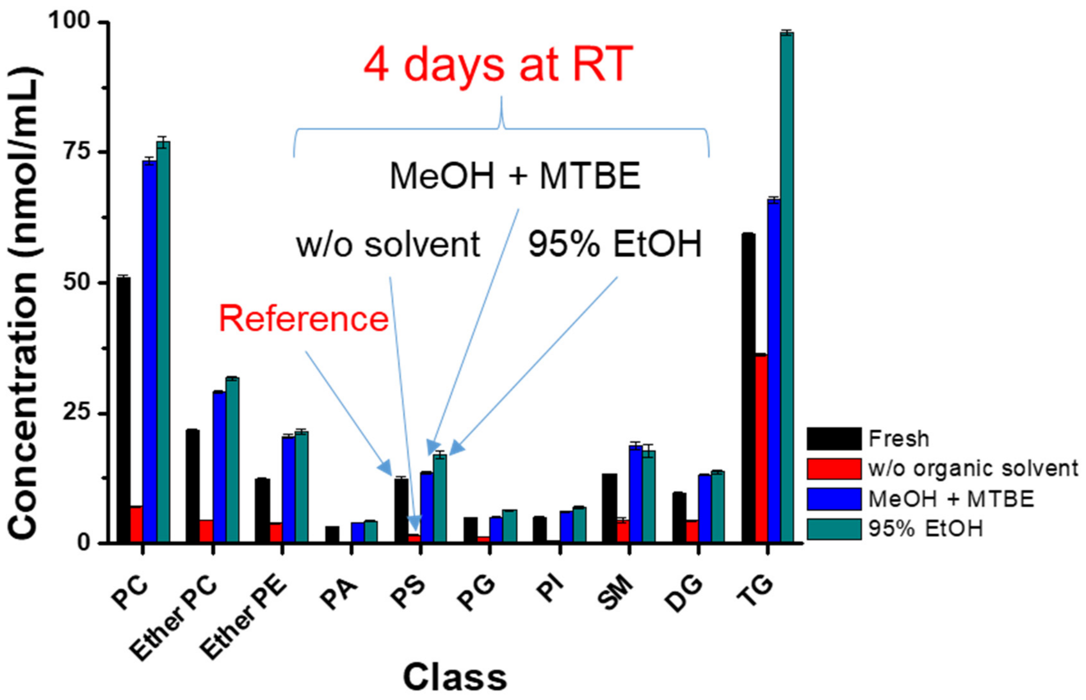

Figure S3. Concentration of each lipid class of saliva upon the immediate extraction after sampling, the exposure to room temperature (RT) with or without adding organic solvents followed by 4 days of exposure to RT. Error bar was obtained from repeated measurements (n = 5).

Table S1. Concentration of lipid species (nmol/mL) in saliva and plasma samples from both the control and the lung cancer groups. Lipid concentration was calculated from a calibration curve of each lipid class. Species marked with underline for high abundance species in each lipid class. Numbers in the parenthesis represent the quantified/identified numbers of lipids. N.D. represents for "not detectable" as below LOD (S/N = 3). Concentration values marked with grey represent for the calculated concentration below LOQ (S/N=10). LC: lung cancer, C: control.

Saliva						
Class	Molecular species	*m/z*	Concentration (nmol/mL) C	LC	abund. (%)	Fold ratio LC/C
LPC	14:0	468.309	0.03 ± 0.03	0.04 ± 0.00	1.2	1.16 ± 1.08
(9)	16:1	494.324	0.08 ± 0.01	0.12 ± 0.01	3.1	1.48 ± 0.25
	16:0	496.340	0.42 ± 0.14	0.45 ± 0.01	17.5	1.00 ± 0.30
	18:2	520.340	0.55 ± 0.08	0.51 ± 0.02	21.3	0.78 ± 0.11
	18:1	522.355	1.02 ± 0.07	0.99 ± 0.02	40.0	0.75 ± 0.06
	18:0	524.371	0.20 ± 0.05	0.20 ± 0.01	8.2	0.93 ± 0.22
	20:4	544.340	0.15 ± 0.05	0.11 ± 0.01	6.2	0.53 ± 0.18**
	20:3	546.355	0.06 ± 0.02	0.04 ± 0.00	2.5	**0.49 ± 0.13****
	22:5	570.355	N.D.	N.D.	-	-
PC	30:0	706.538	0.06 ± 0.01	0.05 ± 0.08	0.8	0.91 ± 0.61
(21)	32:1	732.554	0.29 ± 0.02	0.19 ± 0.09	3.9	0.66 ± 0.15
	32:0	734.569	0.63 ± 0.05	0.32 ± 0.16	8.3	0.52 ± 0.13**
	34:2	758.569	1.14 ± 0.06	0.57 ± 0.13	15.1	**0.50 ± 0.06****
	34:1	760.585	1.27 ± 0.11	0.79 ± 0.17	16.9	0.62 ± 0.08**
	34:0	762.601	0.28 ± 0.04	0.05 ± 0.02	3.7	**0.17 ± 0.04****
	36:4	782.569	0.38 ± 0.03	0.07 ± 0.01	5.0	**0.18 ± 0.02****
	36:3	784.585	0.65 ± 0.01	0.33 ± 0.02	8.7	0.51 ± 0.02**
	36:2	786.601	1.29 ± 0.08	0.54 ± 0.08	17.2	**0.42 ± 0.04****
	36:1	788.616	0.96 ± 0.17	0.40 ± 0.06	12.8	**0.41 ± 0.08****
	36:0	790.632	N.D.	N.D.	-	-
	38:6	806.569	0.07 ± 0.00	N.D.	0.9	-
	38:5	808.585	0.12 ± 0.01	0.04 ± 0.00	1.6	**0.36 ± 0.02****
	38:4	810.601	0.11 ± 0.01	0.04 ± 0.01	1.4	**0.41 ± 0.04****
	38:3	812.616	0.12 ± 0.01	N.D.	1.6	**-**
	38:2	814.632	0.12 ± 0.02	N.D.	1.5	**-**
	38:1	816.648	0.04 ± 0.01	N.D.	0.5	-
	40:6	834.601	N.D.	N.D.	-	-
	40:2	842.663	N.D.	N.D.	-	-
	42:0	874.726	N.D.	N.D.	-	-
	44:1	900.742	N.D.	N.D.	-	-
EtherLPC	O-16:1 and P-16:0	482.361	0.04 ± 0.00	N.D.	5.5	-
(6)	O-16:0	480.345	0.09 ± 0.00	0.03 ± 0.00	14.3	**0.27 ± 0.04****
	O-18:1 and P-18:0	508.376	0.05 ± 0.00	N.D.	7.1	**-**
	O-22:1	564.439	0.11 ± 0.00	0.04 ± 0.00	17.1	**0.39 ± 0.05****
	P-24:1	592.470	0.10 ± 0.00	N.D.	15.5	**-**
	O-24:1	590.455	0.27 ± 0.01	0.15 ± 0.02	40.5	0.55 ± 0.09
EtherPC	O-32:2 and P-32:1	716.559	0.20 ± 0.02	0.14 ± 0.00	1.2	0.71 ± 0.32
(19)	O-32:1 and P-32:0	718.575	0.79 ± 0.01	0.43 ± 0.02	4.8	0.55 ± 0.06**

	O-32:0	720.590	1.78 ± 0.02	0.76 ± 0.05	10.8	**0.43 ± 0.05****
	O-34:3 and P-34:2	742.575	0.49 ± 0.01	0.24 ± 0.01	3.0	**0.49 ± 0.04****
	O-34:2 and P-34:1	744.590	2.05 ± 0.04	0.97 ± 0.05	12.4	**0.47 ± 0.06****
	O-34:1 and P-34:0	746.606	2.96 ± 0.05	0.82 ± 0.05	18.0	**0.28 ± 0.03****
	O-36:4	768.590	0.50 ± 0.00	0.18 ± 0.01	3.0	**0.36 ± 0.03****
	O-36:3 and P-36:2	770.606	0.90 ± 0.02	0.26 ± 0.01	5.5	**0.29 ± 0.03****
	O-36:2 and P-36:1	772.622	2.01 ± 0.05	0.41 ± 0.01	12.2	**0.20 ± 0.03****
	O-36:0	776.653	0.14 ± 0.00	N.D.	0.8	**-**
	O-38:5 and P-38:4	794.606	0.57 ± 0.01	0.16 ± 0.01	3.4	**0.29 ± 0.03****
	O-38:4	796.622	0.65 ± 0.01	0.16 ± 0.01	4.0	**0.24 ± 0.03****
	O-38:3	798.637	0.53 ± 0.01	0.11 ± 0.01	3.2	**0.20 ± 0.03****
	O-38:2	800.653	0.66 ± 0.02	0.10 ± 0.00	4.0	**0.15 ± 0.02****
	O-38:1	802.668	0.38 ± 0.01	0.04 ± 0.00	2.3	**0.12 ± 0.02****
	O-38:0	804.684	0.05 ± 0.00	N.D.	0.3	-
	O-40:3	826.668	0.54 ± 0.02	0.12 ± 0.01	3.3	**0.21 ± 0.03****
	O-40:2 and P-40:1	828.684	0.50 ± 0.02	0.06 ± 0.00	3.0	**0.12 ± 0.02****
	O-42:3 and P-42:2	854.700	0.79 ± 0.02	0.11 ± 0.01	4.8	**0.14 ± 0.02****
LPE	14:0	426.262	0.06 ± 0.01	0.04 ± 0.01	2.1	0.52 ± 0.23*
(13)	16:1	452.277	0.21 ± 0.00	0.15 ± 0.01	6.1	0.73 ± 0.10
	16:0	454.293	0.47 ± 0.00	0.32 ± 0.01	13.2	0.74 ± 0.04
	18:2	478.293	0.57 ± 0.01	0.35 ± 0.01	16.3	0.57 ± 0.08**
	18:1	480.309	1.05 ± 0.04	0.57 ± 0.01	29.6	0.54 ± 0.10**
	18:0	482.324	0.21 ± 0.01	0.21 ± 0.02	5.9	1.33 ± 0.33
	20:4	502.293	0.46 ± 0.02	0.28 ± 0.01	12.6	0.68 ± 0.15
	20:3	504.309	0.04 ± 0.00	0.04 ± 0.01	1.0	1.16 ± 0.47
	20:2	506.324	0.02 ± 0.00	N.D.	0.6	-
	20:1	508.340	0.57 ± 0.01	0.14 ± 0.01	7.9	**0.32 ± 0.06****
	22:6	526.2928	0.07 ± 0.00	0.06 ± 0.00	2.0	1.11 ± 0.11
	22:4	530.324	0.09 ± 0.00	0.04 ± 0.00	2.2	**0.42 ± 0.07****
	24:0	566.418	0.03 ± 0.00	0.02 ± 0.01	0.5	1.09 ± 1.04
PE	30:1	662.476	0.09 ± 0.00	N.D.	3.6	-
(14)	30:0	664.491	0.22 ± 0.00	0.09 ± 0.06	9.1	**0.44 ± 0.05****
	32:2	688.491	0.12 ± 0.00	N.D.	5.2	-
	32:1	690.507	0.25 ± 0.00	0.06 ± 0.06	10.7	**0.25 ± 0.03****
	32:0	692.523	N.D.	N.D.	-	-
	34:3	714.507	0.05 ± 0.00	N.D.	2.0	-
	34:2	716.523	0.31 ± 0.00	0.16 ± 0.07	13.3	0.51 ± 0.04**
	34:1	718.538	0.21 ± 0.01	0.10 ± 0.06	8.8	**0.48 ± 0.09****
	36:4	740.523	0.05 ± 0.00	N.D.	1.9	-
	36:3	742.538	0.14 ± 0.00	N.D.	5.9	-
	36:2	744.554	0.25 ± 0.00	0.11 ± 0.04	10.8	**0.44 ± 0.03****
	36:1	746.569	0.31 ± 0.00	0.34 ± 0.19	13.0	1.10 ± 0.11
	38:5	766.538	N.D.	N.D.	-	-
	38:2	772.585	0.37 ± 0.01	0.28 ± 0.13	15.5	0.76 ± 0.09
EtherLPE	P-16:0	438.298	0.29 ± 0.00	0.24 ± 0.01	35.3	0.83 ± 0.07
(4)	P-18:1	464.314	0.10 ± 0.00	0.08 ± 0.00	11.9	0.76 ± 0.07

	P-18:0	466.329	0.37 ± 0.01	0.21 ± 0.01	<u>44.1</u>	0.57 ± 0.07*
	P-20:0	494.361	0.07 ± 0.00	0.04 ± 0.00	8.7	0.52 ± 0.06*
EtherPE	P-32:1	674.512	0.44 ± 0.01	0.43 ± 0.02	2.4	0.98 ± 0.11
(15)	P-34:2	700.528	0.84 ± 0.05	0.52 ± 0.05	4.6	0.62 ± 0.17*
	P-34:1	702.543	4.46 ± 0.05	2.80 ± 0.21	<u>24.1</u>	0.63 ± 0.06*
	P-34:0	704.559	0.56 ± 0.03	0.43 ± 0.04	3.0	0.77 ± 0.19
	P-36:5	730.575	1.15 ± 0.01	0.59 ± 0.04	6.2	0.52 ± 0.05*
	P-36:2	728.559	2.18 ± 0.03	1.02 ± 0.06	<u>11.7</u>	**0.47 ± 0.04****
	P-36:1	748.528	2.09 ± 0.04	0.77 ± 0.03	<u>11.3</u>	**0.37 ± 0.04****
	P-38:6	750.543	0.46 ± 0.01	0.36 ± 0.02	2.5	0.77 ± 0.08
	P-38:4	752.559	2.33 ± 0.03	1.10 ± 0.05	<u>12.6</u>	**0.47 ± 0.04****
	O-38:4 and P-38:3	754.575	0.80 ± 0.01	0.34 ± 0.02	4.3	**0.43 ± 0.04****
	P-38:2	756.590	0.38 ± 0.01	0.16 ± 0.01	2.0	**0.44 ± 0.05****
	P-38:1	758.606	0.66 ± 0.02	0.34 ± 0.02	3.6	0.51 ± 0.08*
	P-38:0	760.622	0.94 ± 0.02	0.35 ± 0.03	5.1	**0.37 ± 0.05****
	P-40:5	778.575	0.46 ± 0.00	0.18 ± 0.01	2.5	**0.40 ± 0.03****
	P-40:4	780.590	0.78 ± 0.01	0.17 ± 0.02	4.2	**0.22 ± 0.03****
LPA	16:1	407.220	N.D.	N.D.	-	-
(8)	16:0	409.236	0.09 ± 0.01	0.07 ± 0.02	16.9	0.53 ± 0.23*
	18:3	431.220	N.D.	N.D.	-	-
	18:2	433.236	0.06 ± 0.00	0.05 ± 0.01	11.0	0.63 ± 0.17
	18:1	432.252	N.D.	N.D.	-	-
	18:0	437.267	0.47 ± 0.03	0.13 ± 0.03	<u>69.4</u>	**0.30 ± 0.12**
	20:4	457.236	0.02 ± 0.00	N.D.	2.8	**-**
	20:3	459.252	N.D.	N.D.	-	-
PA	30:0	619.434	0.04 ± 0.00	N.D.	1.5	-
(6)	34:2	671.466	0.45 ± 0.01	0.17 ± 0.00	<u>17.1</u>	**0.39 ± 0.03****
	34:1	673.481	0.82 ± 0.01	0.25 ± 0.00	<u>31.0</u>	**0.31 ± 0.02****
	36:3	697.481	0.31 ± 0.00	0.09 ± 0.00	11.6	**0.31 ± 0.02****
	36:2	699.497	0.84 ± 0.01	0.27 ± 0.00	<u>31.8</u>	**0.32 ± 0.03****
	38:4	723.497	0.19 ± 0.01	0.08 ± 0.00	7.1	**0.42 ± 0.09****
LPS	16:1	494.253	0.24 ± 0.01	0.16 ± 0.01	3.6	0.65 ± 0.17
(10)	16:0	496.268	N.D.	N.D.	-	-
	18:2	520.268	1.03 ± 0.03	0.52 ± 0.06	<u>15.5</u>	0.51 ± 0.14**
	18:1	522.284	4.05 ± 0.15	1.52 ± 0.14	<u>61.2</u>	**0.38 ± 0.10****
	18:0	524.299	0.18 ± 0.01	0.10 ± 0.03	2.6	0.57 ± 0.36
	20:4	544.268	0.65 ± 0.02	0.27 ± 0.03	9.8	**0.42 ± 0.10****
	20:3	546.284	0.25 ± 0.01	0.14 ± 0.02	3.8	0.54 ± 0.17*
	22:6	568.268	0.14 ± 0.01	0.10 ± 0.02	2.1	0.70 ± 0.34
	22:5	570.284	N.D.	N.D.	-	-
	22:0	580.362	0.08 ± 0.01	N.D.	1.2	-
PS	30:0	706.467	0.05 ± 0.01	N.D.	0.3	-
(18)	32:2	730.467	0.10 ± 0.01	0.04 ± 0.00	0.6	**0.38 ± 0.22***
	32:1	732.482	0.16 ± 0.01	0.05 ± 0.01	0.9	**0.28 ± 0.15****
	34:2	758.498	0.50 ± 0.02	0.11 ± 0.00	2.7	**0.23 ± 0.05****
	34:1	760.513	1.23 ± 0.06	0.39 ± 0.01	<u>6.6</u>	**0.32 ± 0.08****
	36:3	784.513	0.17 ± 0.01	N.D.	0.9	-
	36:2	786.529	2.45 ± 0.08	0.74 ± 0.02	<u>13.1</u>	**0.30 ± 0.05****
	36:1	788.545	12.02 ± 0.20	2.72 ± 0.10	<u>64.4</u>	**0.23 ± 0.03****
	38:4	810.529	0.51 ± 0.03	0.09 ± 0.00	2.7	**0.17 ± 0.06****
	38:3	812.545	0.38 ± 0.01	0.14 ± 0.00	2.1	**0.35 ± 0.05****
	38:2	814.560	0.27 ± 0.00	0.07 ± 0.00	1.5	**0.26 ± 0.03****

	38:1	816.576	0.06 ± 0.01	0.08 ± 0.00	0.3	1.36 ± 0.99
	40:6	834.529	0.17 ± 0.00	0.10 ± 0.01	0.9	0.57 ± 0.10*
	40:2	842.592	0.14 ± 0.00	0.04 ± 0.00	0.8	0.27 ± 0.04*
	40:1	844.607	0.13 ± 0.01	0.06 ± 0.00	0.7	**0.45 ± 0.11****
	42:3	868.607	N.D.	N.D.	-	-
	42:2	870.623	0.08 ± 0.00	N.D.	0.4	-
	42:1	872.639	0.23 ± 0.02	0.04 ± 0.00	1.2	**0.16 ± 0.05****
LPG	14:1	453.226	N.D.	N.D.	-	-
(9)	14:0	455.242	N.D.	N.D.	-	-
	16:1	481.257	N.D.	N.D.	-	-
	16:0	483.273	0.10 ± 0.00	0.05 ± 0.03	33.1	0.54 ± 0.14*
	18:2	507.273	0.03 ± 0.00	0.02 ± 0.01	9.4	0.56 ± 0.25*
	18:1	509.289	0.13 ± 0.02	0.06 ± 0.03	44.2	**0.44 ± 0.25***
	18:0	511.304	0.04 ± 0.01	0.02 ± 0.02	13.2	0.53 ± 0.58*
	20:3	533.289	N.D.	N.D.	-	-
	20:1	537.320	N.D.	N.D.	-	-
PG	30:1	691.456	N.D.	N.D.	-	-
(19)	30:0	693.471	0.08 ± 0.03	0.05 ± 0.00	3.4	0.66 ± 0.98
	32:1	719.487	0.22 ± 0.23	0.07 ± 0.00	9.4	**0.30 ± 1.49****
	32:0	721.503	0.14 ± 0.08	0.13 ± 0.01	5.9	0.96 ± 2.56
	34:3	743.487	0.04 ± 0.08	N.D.	1.5	-
	34:2	745.503	0.25 ± 0.07	0.09 ± 0.01	10.7	**0.37 ± 0.52****
	34:1	747.518	0.60 ± 0.23	0.31 ± 0.03	25.7	0.52 ± 0.95*
	34:0	749.534	0.15 ± 0.14	0.03 ± 0.00	6.5	**0.20 ± 0.82****
	36:4	769.503	0.09 ± 0.14	0.04 ± 0.00	4.0	**0.38 ± 2.73****
	36:3	771.518	0.20 ± 0.08	0.08 ± 0.01	8.6	**0.38 ± 0.74****
	36:2	773.534	0.46 ± 0.16	0.21 ± 0.04	19.7	**0.46 ± 0.77****
	36:1	775.550	0.11 ± 0.02	0.08 ± 0.02	4.6	0.75 ± 0.81
	36:0	777.565	N.D.	N.D.	-	-
	38:5	795.518	N.D.	N.D.	-	-
	38:4	797.534	N.D.	N.D.	-	-
	38:3	799.550	N.D.	N.D.	-	-
	38:2	801.565	N.D.	N.D.	-	-
	40:7	819.518	N.D.	N.D.	-	-
	40:6	821.534	N.D.	N.D.	-	-
LPI	16:1	569.273	0.54 ± 0.03	0.75 ± 0.00	3.4	1.40 ± 0.34
(8)	16:0	571.289	2.63 ± 0.47	1.87 ± 0.01	16.6	0.71 ± 0.60
	18:2	595.289	1.48 ± 0.10	1.36 ± 0.00	9.4	0.92 ± 0.31
	18:1	597.305	3.78 ± 0.42	1.71 ± 0.01	23.9	0.45 ± 0.24*
	18:0	599.320	6.00 ± 0.31	3.24 ± 0.01	37.9	0.54 ± 0.14**
	20:4	619.289	0.98 ± 0.08	0.57 ± 0.00	6.2	0.59 ± 0.23
	20:3	621.305	0.42 ± 0.01	0.21 ± 0.00	2.7	0.51 ± 0.12*
	22:5	645.305	N.D.	N.D.	-	-
PI	28:0	753.456	0.01 ± 0.00	0.00 ± 0.00	0.1	0.56 ± 0.43
(22)	30:0	781.487	0.03 ± 0.01	0.02 ± 0.00	0.4	0.72 ± 0.67
	32:2	805.487	0.06 ± 0.00	0.04 ± 0.00	0.7	0.56 ± 0.08**
	32:1	807.503	0.27 ± 0.01	0.21 ± 0.00	2.9	0.77 ± 0.13
	32:0	809.519	0.18 ± 0.05	0.10 ± 0.00	1.9	0.56 ± 0.75*
	34:3	831.503	0.08 ± 0.00	0.06 ± 0.00	0.8	0.79 ± 0.08
	34:2	833.519	0.61 ± 0.01	0.65 ± 0.01	6.5	1.06 ± 0.12
	34:1	835.534	1.25 ± 0.04	0.96 ± 0.03	13.3	0.77 ± 0.13
	34:0	837.550	0.37 ± 0.03	0.13 ± 0.01	3.9	**0.35 ± 0.12****
	36:4	857.519	0.15 ± 0.00	0.13 ± 0.00	1.6	0.86 ± 0.10

	36:3	859.534	0.42 ± 0.00	0.26 ± 0.00	4.5	0.63 ± 0.04
	36:2	861.550	1.55 ± 0.06	1.09 ± 0.07	16.5	0.70 ± 0.16
	36:1	863.566	0.99 ± 0.04	0.53 ± 0.02	10.5	0.54 ± 0.11
	36:0	865.581	0.02 ± 0.00	0.01 ± 0.00	0.2	**0.28 ± 0.26****
	38:6	881.519	0.02 ± 0.00	0.01 ± 0.00	0.2	0.76 ± 0.22
	38:5	883.534	0.12 ± 0.00	0.06 ± 0.00	1.3	0.49 ± 0.05*
	38:4	885.550	2.09 ± 0.07	0.99 ± 0.03	22.3	**0.47 ± 0.08****
	38:3	887.566	0.95 ± 0.03	0.51 ± 0.03	10.1	0.54 ± 0.11*
	38:2	889.581	0.07 ± 0.00	0.07 ± 0.00	0.8	0.94 ± 0.31
	38:1	891.597	0.01 ± 0.00	0.01 ± 0.00	0.1	0.68 ± 0.39
	40:5	911.566	0.05 ± 0.00	0.03 ± 0.00	0.5	0.64 ± 0.09
	40:4	913.581	0.07 ± 0.00	0.06 ± 0.00	0.8	0.83 ± 0.16
SM	d32:1	675.544	1.55 ± 0.03	0.87 ± 0.03	4.5	0.56 ± 0.06**
(16)	d32:0	677.559	0.27 ± 0.01	0.23 ± 0.01	0.8	0.84 ± 0.09
	d34:2	701.559	1.02 ± 0.02	0.57 ± 0.02	3.0	0.55 ± 0.07**
	d34:1	703.575	8.86 ± 0.12	8.09 ± 0.24	25.7	0.91 ± 0.08
	d34:0	705.591	6.83 ± 0.16	3.82 ± 0.17	19.8	0.56 ± 0.08**
	d36:2	729.591	0.28 ± 0.01	0.10 ± 0.01	0.8	**0.37 ± 0.06****
	d36:1	731.606	1.00 ± 0.02	0.46 ± 0.02	2.9	**0.46 ± 0.06****
	d36:0	733.622	1.30 ± 0.03	0.63 ± 0.02	3.8	**0.49 ± 0.06****
	d38:2	757.622	0.10 ± 0.00	N.D.	0.3	-
	d38:1	759.637	0.75 ± 0.04	0.36 ± 0.01	2.2	**0.48 ± 0.11****
	d40:2	785.653	0.57 ± 0.02	0.18 ± 0.01	1.6	**0.32 ± 0.06****
	d40:1	787.669	1.67 ± 0.10	0.63 ± 0.02	4.8	**0.38 ± 0.11****
	d40:0	789.684	1.20 ± 0.06	0.33 ± 0.01	3.5	**0.28 ± 0.07****
	d42:2	813.684	5.36 ± 0.23	1.98 ± 0.06	15.5	**0.37 ± 0.08****
	d42:1	815.700	2.54 ± 0.14	0.87 ± 0.04	7.3	**0.34 ± 0.09****
	d42:0	817.716	1.21 ± 0.07	0.34 ± 0.02	3.5	**0.28 ± 0.08****
Cer	d34:2	536.504	0.11 ± 0.01	0.56 ± 0.10	2.3	**5.17 ± 1.29****
(15)	d34:1	538.519	0.59 ± 0.04	1.09 ± 0.42	12.9	1.83 ± 0.71*
	d34:0	540.535	0.54 ± 0.02	1.00 ± 0.55	11.7	1.85 ± 0.62*
	d36:2	564.535	0.04 ± 0.00	0.08 ± 0.04	0.9	1.90 ± 0.46*
	d36:1	566.551	0.24 ± 0.01	0.37 ± 0.09	5.2	1.56 ± 0.29
	d36:0	568.566	0.34 ± 0.01	0.28 ± 0.11	7.3	0.84 ± 0.19
	d38:1	594.582	0.20 ± 0.01	0.19 ± 0.08	4.3	0.97 ± 0.22
	d38:0	596.598	0.29 ± 0.00	0.08 ± 0.05	6.3	**0.27 ± 0.08****
	d40:1	622.613	0.21 ± 0.00	0.22 ± 0.05	4.6	1.03 ± 0.12
	d40:0	624.629	0.19 ± 0.00	0.14 ± 0.06	4.1	0.76 ± 0.16
	d42:2	648.629	0.48 ± 0.01	0.48 ± 0.08	10.3	1.01 ± 0.10
	d42:1	650.645	0.49 ± 0.01	0.28 ± 0.04	10.7	0.58 ± 0.05*
	d42:0	652.660	0.31 ± 0.01	0.46 ± 0.16	6.8	1.46 ± 0.35
	d44:1	678.676	0.27 ± 0.01	0.27 ± 0.13	5.8	1.02 ± 0.24
	d44:0	680.692	0.30 ± 0.00	0.21 ± 0.04	6.6	0.69 ± 0.07
HexCer	d34:1	862.625	0.36 ± 0.04	0.05 ± 0.01	20.8	**0.14 ± 0.07****
(4)	d40:0	946.719	0.33 ± 0.01	0.04 ± 0.01	19.3	**0.13 ± 0.03****
	d42:2	972.735	0.48 ± 0.02	0.06 ± 0.02	28.0	**0.13 ± 0.03****
	d42:0	974.750	0.55 ± 0.02	0.07 ± 0.01	31.9	**0.12 ± 0.03****
Hex2Cer	d34:1	862.625	1.31 ± 0.02	0.76 ± 0.06	32.0	0.58 ± 0.06
(4)	d40:1	946.719	0.32 ± 0.01	0.05 ± 0.01	7.8	**0.14 ± 0.03****
	d42:2	972.735	1.96 ± 0.09	0.49 ± 0.06	47.6	**0.25 ± 0.06****
	d42:1	974.750	0.52 ± 0.02	0.11 ± 0.02	12.7	**0.22 ± 0.05****
DG	30:0	558.509	0.76 ± 0.02	2.67 ± 0.12	1.4	**3.52 ± 0.84****
(14)	32:2	582.509	0.30 ± 0.01	1.73 ± 0.05	0.6	**5.71 ± 1.29****

	32:1	584.525	1.61 ± 0.02	5.74 ± 0.19	3.0	**3.57 ± 0.56****
	32:0	586.541	5.19 ± 0.06	14.19 ± 0.41	9.7	**2.73 ± 0.38****
	34:2	610.541	4.94 ± 0.06	14.60 ± 0.46	9.2	**2.95 ± 0.45****
	34:1	612.556	7.05 ± 0.08	25.83 ± 0.78	13.1	**3.67 ± 0.53****
	34:0	614.572	5.63 ± 0.11	10.28 ± 0.52	10.5	1.82 ± 0.44**
	36:4	634.541	1.94 ± 0.04	10.01 ± 0.34	3.6	**5.16 ± 0.93****
	36:3	636.556	4.46 ± 0.04	8.84 ± 0.22	8.3	1.98 ± 0.23**
	36:2	638.572	8.89 ± 0.12	16.84 ± 0.58	16.6	1.89 ± 0.31*
	36:1	640.588	2.70 ± 0.05	4.38 ± 0.28	5.0	1.62 ± 0.48*
	36:0	642.603	4.91 ± 0.09	2.77 ± 0.09	9.1	0.57 ± 0.10**
	38:4	662.572	3.38 ± 0.07	1.78 ± 0.08	6.3	0.52 ± 0.12**
	38:3	664.588	1.88 ± 0.03	1.25 ± 0.04	3.5	0.66 ± 0.10
TG	38:0	684.614	1.07 ± 0.18	13.47 ± 5.61	1.2	**12.62 ± 17.76****
(43)	40:0	712.645	0.55 ± 0.03	5.41 ± 0.81	0.6	**9.75 ± 4.92****
	42:2	736.645	0.37 ± 0.01	2.25 ± 0.57	0.4	**6.08 ± 4.46**
	42:1	738.661	0.72 ± 0.02	2.11 ± 0.33	0.8	**2.91 ± 1.36**
	42:0	740.676	0.77 ± 0.02	3.14 ± 0.38	0.8	**4.07 ± 1.47****
	44:3	762.661	0.29 ± 0.01	0.80 ± 0.23	0.3	**2.78 ± 2.27**
	44:2	764.676	0.90 ± 0.01	1.94 ± 0.13	1.0	**2.16 ± 0.45***
	44:1	766.692	1.00 ± 0.04	4.47 ± 0.99	1.1	**4.49 ± 2.90****
	44:0	768.708	0.43 ± 0.01	2.49 ± 0.23	0.5	**5.79 ± 1.70****
	46:3	790.692	0.61 ± 0.01	1.06 ± 0.21	0.7	1.75 ± 1.00
	46:2	792.708	1.46 ± 0.03	3.33 ± 0.32	1.6	**2.27 ± 0.67***
	46:1	794.723	1.28 ± 0.03	4.34 ± 0.36	1.4	**3.39 ± 0.88****
	46:0	796.739	0.59 ± 0.02	1.67 ± 0.25	0.6	**2.80 ± 1.26****
	48:3	818.723	1.28 ± 0.03	1.12 ± 0.18	1.4	0.87 ± 0.41
	48:2	820.739	2.07 ± 0.03	3.77 ± 0.29	2.2	1.82 ± 0.41*
	48:1	822.755	1.81 ± 0.01	6.07 ± 0.87	2.0	**3.35 ± 1.35****
	48:0	824.770	0.59 ± 0.01	1.66 ± 0.27	0.6	**2.83 ± 1.32****
	50:3	846.755	3.98 ± 0.04	7.30 ± 0.32	4.3	1.83 ± 0.24**
	50:2	848.770	4.63 ± 0.05	14.01 ± 1.49	5.0	**3.02 ± 0.92****
	50:1	850.786	1.82 ± 0.06	6.21 ± 2.09	2.0	**3.41 ± 3.27****
	50:0	852.802	0.32 ± 0.01	0.37 ± 0.11	0.3	1.16 ± 0.96
	52:6	868.739	0.54 ± 0.02	0.72 ± 0.03	0.6	1.32 ± 0.25
	52:5	870.755	2.78 ± 0.05	9.98 ± 1.27	3.0	**3.59 ± 1.32****
	52:4	872.770	8.52 ± 0.11	19.45 ± 0.76	9.2	**2.28 ± 0.29***
	52:3	874.786	8.52 ± 0.13	23.60 ± 2.00	9.2	**2.77 ± 0.69****
	52:2	876.802	5.04 ± 0.18	21.61 ± 3.16	5.5	**4.29 ± 1.90****
	52:1	878.817	0.98 ± 0.06	2.62 ± 0.76	1.1	**2.68 ± 2.32****
	54:8	892.739	0.11 ± 0.01	0.01 ± 0.01	0.1	**0.08 ± 0.28***
	54:7	894.755	0.98 ± 0.05	1.56 ± 0.09	1.1	1.59 ± 0.44
	54:6	896.770	6.83 ± 0.23	16.67 ± 0.80	7.4	**2.44 ± 0.51***
	54:5	898.786	9.58 ± 0.16	17.72 ± 0.78	10.4	1.85 ± 0.27*
	54:4	900.802	8.61 ± 0.17	20.75 ± 1.01	9.3	**2.41 ± 0.40****
	54:3	902.817	5.25 ± 0.28	14.97 ± 1.32	5.7	**2.85 ± 1.00****
	54:2	904.833	1.25 ± 0.10	2.87 ± 0.63	1.4	**2.30 ± 1.65****
	54:0	906.849	0.22 ± 0.01	0.32 ± 0.17	0.2	1.44 ± 2.25
	56:8	920.770	0.70 ± 0.01	0.31 ± 0.15	0.8	0.43 ± 0.58*
	56:7	922.786	1.50 ± 0.03	1.01 ± 0.26	1.6	0.68 ± 0.49
	56:6	924.802	1.70 ± 0.02	0.21 ± 0.04	1.8	**0.12 ± 0.07****
	56:5	926.817	1.12 ± 0.04	0.68 ± 0.05	1.2	0.61 ± 0.15*
	56:4	928.833	0.56 ± 0.04	0.40 ± 0.03	0.6	0.71 ± 0.25
	56:3	930.849	0.27 ± 0.02	0.17 ± 0.02	0.3	0.64 ± 0.30

	56:2	932.864	0.11 ± 0.01	0.09 ± 0.02	0.1	0.87 ± 0.57
	58:8	948.801	0.43 ± 0.01	0.08 ± 0.01	0.5	**0.19 ± 0.08****

Plasma

Class	Molecular species	*m/z*	Concentration (nmol/mL) C	LC	abund. (%)	Fold ratio LC/C
LPC (14)	14:0	468.5	3.13 ± 0.56	3.05 ± 0.40	1.9	0.98 ± 0.22
	16:1	494.5	3.89 ± 0.66	3.74 ± 0.65	2.4	0.96 ± 0.23
	16:0	496.5	63.93 ± 8.77	58.49 ± 7.09	39.0	0.91 ± 0.17
	18:2	520.5	49.70 ± 6.79	30.31 ± 5.00	30.3	0.61 ± 0.13**
	18:1	522.5	16.13 ± 1.99	11.69 ± 0.93	9.8	0.72 ± 0.11**
	18:0	524.5	9.78 ± 2.21	8.02 ± 1.11	6.0	0.82 ± 0.22*
	20:4	544.5	10.68 ± 1.70	8.35 ± 1.52	6.5	0.78 ± 0.19**
	20:3	546.5	3.12 ± 0.42	2.40 ± 0.37	1.9	0.77 ± 0.16**
	20:2	548.5	0.42 ± 0.10	0.34 ± 0.07	0.3	0.81 ± 0.26**
	20:1	550.5	N.D.	N.D.	-	-
	20:0	552.5	0.28 ± 0.08	0.23 ± 0.05	0.2	0.82 ± 0.29
	22:6	568.5	1.99 ± 0.36	1.35 ± 0.26	1.2	0.68 ± 0.18**
	22:5	570.5	0.46 ± 0.09	0.33 ± 0.07	0.3	0.72 ± 0.20**
	22:4	572.5	0.28 ± 0.08	N.D.	0.2	-
EtherLPC (3)	O-16:0	480.5	1.10 ± 0.24	1.09 ± 0.21	57.6	0.99 ± 0.29
	O-16:1 and P-16:0	482.5	N.D.	N.D.	-	-
	O-18:0	510.5	0.81 ± 0.22	0.93 ± 0.18	42.4	1.15 ± 0.38
PC (25)	30:0	706.5	1.34 ± 0.27	1.18 ± 0.29	0.3	0.87 ± 0.28
	32:2	730.5	3.14 ± 0.61	2.00 ± 0.37	0.7	0.64 ± 0.17**
	32:1	732.5	5.38 ± 1.14	5.17 ± 0.76	1.2	0.96 ± 0.25
	32:0	734.5	2.83 ± 0.67	2.95 ± 0.48	0.6	1.04 ± 0.30
	34:4	754.5	1.28 ± 0.26	0.89 ± 0.17	0.3	0.70 ± 0.19**
	34:3	756.5	5.66 ± 0.78	3.44 ± 0.55	1.3	0.61 ± 0.13**
	34:2	758.5	114.82 ± 16.89	85.66 ± 7.79	25.8	0.75 ± 0.13**
	34:1	760.5	50.63 ± 8.12	50.21 ± 7.65	11.4	0.99 ± 0.22
	34:0	762.5	5.23 ± 0.86	5.64 ± 0.82	1.2	1.08 ± 0.24
	36:6	778.5	0.27 ± 0.11	0.17 ± 0.07	0.1	0.64 ± 0.36*
	36:4	782.5	53.28 ± 7.90	32.14 ± 5.07	12.0	0.60 ± 0.13**
	36:3	784.5	39.88 ± 4.45	26.92 ± 3.93	9.0	0.67 ± 0.12**
	36:2	786.5	59.58 ± 9.46	43.05 ± 7.16	13.4	0.72 ± 0.17**
	36:1	788.5	18.04 ± 3.20	15.99 ± 3.07	4.0	0.89 ± 0.23
	36:0	790.5	1.98 ± 0.47	1.74 ± 0.36	0.4	0.88 ± 0.28
	38:7	804.5	0.64 ± 0.17	0.52 ± 0.11	0.1	0.82 ± 0.28
	38:6	806.5	17.15 ± 2.35	10.09 ± 1.75	3.8	0.59 ± 0.13**
	38:5	808.5	12.09 ± 1.60	7.61 ± 1.58	2.7	0.63 ± 0.15**
	38:4	810.5	20.98 ± 3.81	16.23 ± 2.45	4.7	0.77 ± 0.18**
	38:3	812.5	14.87 ± 3.13	13.97 ± 2.58	3.3	0.94 ± 0.26
	38:2	814.5	8.74 ± 1.77	9.78 ± 2.16	2.0	1.12 ± 0.34
	40:8	830.5	0.77 ± 0.18	0.60 ± 0.17	0.2	0.79 ± 0.29**
	40:7	832.5	1.26 ± 0.23	0.71 ± 0.21	0.3	0.57 ± 0.19**
	40:6	834.5	3.85 ± 0.68	2.77 ± 0.33	0.9	0.72 ± 0.15**
	40:5	836.5	1.90 ± 0.34	1.66 ± 0.35	0.4	0.87 ± 0.24
EtherPC (19)	P-32:0	718.5	1.58 ± 0.27	1.66 ± 0.35	2.1	1.06 ± 0.28
	O-32:0	720.5	1.77 ± 0.32	2.24 ± 0.46	2.3	1.26 ± 0.34**
	O-34:3	742.5	3.70 ± 0.67	2.79 ± 0.55	4.9	0.75 ± 0.20**
	O-34:2 and P-34:1	744.5	6.27 ± 1.09	5.76 ± 1.01	8.3	0.92 ± 0.23

	O-34:1	746.5	4.67 ± 1.04	6.13 ± 1.28	6.1	1.31 ± 0.40**
	O-34:0	748.5	1.15 ± 0.32	1.54 ± 0.33	1.5	1.33 ± 0.46**
	P-36:4	766.5	5.73 ± 0.95	4.24 ± 0.84	7.5	0.74 ± 0.19**
	O-36:4 and P-36:3	768.5	9.87 ± 1.37	7.03 ± 1.32	13.0	0.71 ± 0.17**
	O-36:3 and P-36:2	770.5	4.91 ± 0.91	4.55 ± 0.84	6.5	0.93 ± 0.24
	O-36:2 and P-36:1	772.5	7.65 ± 1.30	7.92 ± 1.21	10.1	1.03 ± 0.24
	P-38:5	792.5	3.10 ± 0.52	2.38 ± 0.61	4.1	0.77 ± 0.23**
	O-38:5 and P-38:4	794.5	9.08 ± 1.04	7.00 ± 1.57	12.0	0.77 ± 0.19**
	O-38:4	796.5	7.97 ± 1.18	7.73 ± 1.26	10.5	0.97 ± 0.21
	O-38:3	798.5	2.71 ± 0.38	4.91 ± 0.94	3.6	1.81 ± 0.43**
	O-40:6 and P-40:5	820.5	1.84 ± 0.34	1.82 ± 0.39	2.4	0.99 ± 0.28
	O-40:5 and P-40:4	822.5	1.61 ± 0.26	2.01 ± 0.54	2.1	1.25 ± 0.39**
	O-40:4	824.5	1.18 ± 0.29	1.77 ± 0.54	1.6	1.50 ± 0.59**
	O-42:6	848.5	0.56 ± 0.14	0.54 ± 0.19	0.7	0.97 ± 0.41
	O-42:5	850.5	0.54 ± 0.16	0.94 ± 0.25	0.7	1.75 ± 0.71**
LPE	14:0	426.5	N.D.	N.D.	-	-
(9)	16:1	452.5	N.D.	N.D.	-	-
	16:0	454.5	0.48 ± 0.21	0.44 ± 0.22	4.9	0.91 ± 0.60
	18:2	478.5	4.29 ± 1.45	2.49 ± 1.06	43.6	0.58 ± 0.31**
	18:1	480.5	0.80 ± 0.34	0.49 ± 0.23	8.1	0.62 ± 0.39**
	18:0	482.5	0.55 ± 0.30	0.48 ± 0.30	5.6	0.87 ± 0.72
	20:4	502.5	2.39 ± 0.61	1.79 ± 0.66	24.3	0.75 ± 0.33**
	22:6	526.5	1.13 ± 0.44	0.80 ± 0.42	11.5	0.71 ± 0.46*
	22:5	528.5	0.20 ± 0.10	N.D.	2.1	-
PE	34:2	716.5	2.67 ± 0.55	2.65 ± 1.06	7.2	0.99 ± 0.45
(13)	34:1	718.5	1.17 ± 0.40	1.13 ± 0.50	3.2	0.97 ± 0.54
	36:4	740.5	3.59 ± 0.67	3.01 ± 1.34	9.7	0.84 ± 0.40
	36:3	742.5	3.16 ± 0.78	1.84 ± 0.85	8.6	0.58 ± 0.30**
	36:2	744.5	5.83 ± 1.45	3.63 ± 1.00	15.8	0.62 ± 0.23**
	36:1	746.5	1.67 ± 0.60	1.45 ± 0.62	4.5	0.86 ± 0.48
	38:6	764.5	4.23 ± 1.67	2.74 ± 0.96	11.4	0.65 ± 0.34**
	38:5	766.5	2.80 ± 0.71	1.95 ± 0.93	7.6	0.70 ± 0.37**
	38:4	768.5	7.23 ± 1.95	4.28 ± 1.85	19.6	0.59 ± 0.30**
	38:3	770.5	1.55 ± 0.52	0.96 ± 0.37	4.2	0.62 ± 0.32**
	40:7	790.5	0.53 ± 0.24	0.30 ± 0.17	1.4	0.55 ± 0.40**
	40:6	792.5	1.72 ± 0.69	1.09 ± 0.49	4.7	0.63 ± 0.38**
	40:5	794.5	0.77 ± 0.29	0.42 ± 0.19	2.1	0.54 ± 0.33**
EtherPE	O-36:4 and P-36:3	726.5	0.54 ± 0.23	0.41 ± 0.27	12.8	0.76 ± 0.60
(7)	P-36:2	728.5	1.41 ± 0.42	1.45 ± 0.56	33.4	1.03 ± 0.51
	O-36:2 and P-36:1	730.5	0.28 ± 0.13	0.30 ± 0.15	6.7	1.06 ± 0.75
	O-38:5	752.5	0.94 ± 0.28	0.81 ± 0.45	22.4	0.86 ± 0.54
	O-38:4 and P-38:3	754.5	0.77 ± 0.34	0.62 ± 0.22	18.4	0.81 ± 0.46
	P-38:1	758.5	0.27 ± 0.10	0.16 ± 0.08	6.3	0.61 ± 0.38**
	P-40:7	774.5	N.D.	N.D.	-	-
LPG	14:0	455.5	N.D.	N.D.	-	-
(6)	16:1	481.5	N.D.	N.D.	-	-

	16:0	483.5	N.D.	N.D.	-	-
	18:2	507.5	0.18 ± 0.03	0.17 ± 0.04	42.0	0.95 ± 0.27
	18:1	509.5	0.24 ± 0.06	0.17 ± 0.05	58.0	0.69 ± 0.26**
	18:0	511.5	N.D.	N.D.	-	-
PG	16:0/16:0	721.5	0.34 ± 0.02	N.D.	100	-
(7)	16:0/18:1	747.5	N.D.	N.D.	-	-
	18:0/16:1	747.5	N.D.	N.D.	-	-
	18:1/18:2	771.5	N.D.	N.D.	-	-
	18:0/18:2	773.5	N.D.	N.D.	-	-
	18:1/18:1	773.5	N.D.	N.D.	-	-
	18:0/18:1	775.5	N.D.	N.D.	-	-
	16:0/22:5	795.5	N.D.	N.D.	-	-
LPI	18:2	595.5	2.75 ± 0.81	1.77 ± 0.61	35.8	0.64 ± 0.29**
(5)	18:1	597.5	0.87 ± 0.13	0.66 ± 0.15	11.3	0.76 ± 0.21**
	18:0	599.5	0.49 ± 0.18	0.35 ± 0.11	6.4	0.71 ± 0.35*
	20:4	619.5	2.95 ± 0.22	2.71 ± 0.66	38.5	0.92 ± 0.23
	20:3	621.5	0.61 ± 0.16	0.53 ± 0.15	8.0	0.86 ± 0.34
PI	16:0/16:0	809.5	N.D.	N.D.	-	-
(13)	16:1/18:1	833.5	N.D.	N.D.	-	-
	18:1/16:0	835.5	4.22 ± 1.31	4.79 ± 2.33	4.7	1.13 ± 0.65
	16:0/20:5	855.5	0.33 ± 0.10	0.31 ± 0.19	0.4	0.94 ± 0.65
	18:1/18:2	859.5	2.22 ± 1.18	0.69 ± 0.18	2.5	**0.31 ± 0.18****
	18:0/18:2	861.5	14.60 ± 2.60	7.80 ± 2.42	16.3	0.53 ± 0.19**
	18:1/18:1	861.5	6.08 ± 2.14	3.19 ± 1.20	6.8	0.52 ± 0.27**
	18:1/18:0	863.5	5.02 ± 1.28	2.78 ± 0.81	5.6	0.55 ± 0.22**
	22:6/16:0	881.5	N.D.	N.D.	-	-
	18:0/20:4	885.5	47.32 ± 6.60	23.13 ± 3.76	53.0	**0.49 ± 0.10****
	18:0/20:3	887.5	8.88 ± 1.48	6.01 ± 1.04	9.9	0.68 ± 0.16**
	18:0/20:2	889.5	0.69 ± 0.36	0.25 ± 0.05	0.8	**0.36 ± 0.20****
	18:1/22:6	907.5	N.D.	N.D.	-	-
SM	d18:2/14:0	673.5	1.17 ± 0.36	0.72 ± 0.32	0.4	0.62 ± 0.33**
(8)	d18:1/16:0	703.5	86.13 ± 12.41	84.28 ± 12.77	29.6	0.98 ± 0.20
	d18:2/18:0	729.5	7.55 ± 1.17	7.67 ± 1.30	2.6	1.02 ± 0.23
	d18:1/18:0	731.5	17.41 ± 3.02	19.88 ± 4.75	6.0	1.14 ± 0.34
	d18:0/20:2	757.5	12.68 ± 2.25	9.85 ± 2.31	4.4	0.78 ± 0.23**
	d18:1/22:1	785.5	80.94 ± 13.97	63.29 ± 13.99	27.9	0.78 ± 0.22**
	d18:1/24:1	813.5	60.33 ± 12.49	67.31 ± 15.72	20.8	1.12 ± 0.35
	d18:1/24:0	815.5	24.38 ± 5.74	24.69 ± 6.95	8.4	1.01 ± 0.37
Cer	d16:1/24:0	622.5	1.17 ± 0.49	0.05 ± 0.01	2.5	**0.05 ± 0.02****
(8)	d18:1/22:0	622.5	7.68 ± 0.95	0.91 ± 0.14	16.4	**0.12 ± 0.02****
	d18:0/22:0	624.5	0.38 ± 0.20	N.D.	0.8	-
	d18:1/24:1	648.5	8.20 ± 2.79	1.11 ± 0.11	17.5	**0.14 ± 0.05****
	d18:2/24:0	648.5	4.69 ± 0.78	0.35 ± 0.13	10.0	**0.07 ± 0.03****
	d18:1/24:0	650.5	24.36 ± 5.85	2.14 ± 0.74	52.0	**0.09 ± 0.04****
	d18:1/26:0	678.5	0.21 ± 0.15	N.D.	0.5	-
	d20:1/24:0	678.5	0.20 ± 0.16	N.D.	0.4	-
HexCer	d18:1/22:0	784.5	3.86 ± 1.92	2.10 ± 0.68	33.3	0.55 ± 0.32**
(3)	d18:1/24:1	810.5	3.32 ± 1.68	2.52 ± 1.02	28.7	0.76 ± 0.49
	d18:1/24:0	812.5	4.39 ± 2.50	3.20 ± 2.09	37.9	0.73 ± 0.63
DG	32:1	584.5	0.86 ± 0.63	0.48 ± 0.32	2.7	0.56 ± 0.55*
(10)	32:0	586.5	2.66 ± 2.57	1.62 ± 0.80	8.5	0.61 ± 0.66
	34:2	610.5	2.75 ± 1.50	2.26 ± 1.06	8.8	0.82 ± 0.59
	34:1	612.5	4.50 ± 2.33	4.83 ± 2.10	14.4	1.07 ± 0.72

	34:0	614.5	1.22 ± 0.73	1.04 ± 0.58	3.9	0.85 ± 0.70
	36:4	634.5	0.30 ± 0.19	N.D.	1.0	-
	36:3	636.5	5.99 ± 2.27	4.92 ± 2.35	<u>19.2</u>	0.82 ± 0.50*
	36:2	638.5	11.75 ± 3.47	12.18 ± 6.29	<u>37.6</u>	1.04 ± 0.62
	36:1	640.5	0.89 ± 0.25	1.19 ± 0.65	2.9	1.33 ± 0.82
	36:0	642.5	0.33 ± 0.03	0.31 ± 0.11	1.1	0.96 ± 0.35
TG	40:1	710.5	0.09 ± 0.04	0.21 ± 0.10	0.0	**2.26 ± 1.41***
(42)	40:0	712.5	0.16 ± 0.11	0.79 ± 0.29	0.1	**5.09 ± 3.98****
	42:2	736.5	N.D.	0.42 ± 0.15	-	-
	42:1	738.5	0.10 ± 0.06	0.68 ± 0.22	0.1	**6.53 ± 4.55****
	42:0	740.5	0.47 ± 0.26	1.28 ± 0.34	0.3	**2.73 ± 1.69****
	44:2	764.5	0.11 ± 0.02	0.48 ± 0.24	0.1	**4.22 ± 2.17***
	44:1	766.5	0.66 ± 0.45	0.98 ± 0.33	0.4	1.48 ± 1.13
	44:0	768.5	1.28 ± 0.60	1.40 ± 0.74	0.7	1.10 ± 0.78
	46:3	790.5	0.30 ± 0.06	0.87 ± 0.44	0.2	**2.93 ± 1.58****
	46:2	792.5	1.09 ± 0.69	1.41 ± 1.01	0.6	1.29 ± 1.23
	46:1	794.5	2.46 ± 1.46	3.59 ± 0.66	1.3	1.46 ± 0.91
	46:0	796.5	2.30 ± 1.33	2.19 ± 1.08	1.2	0.95 ± 0.72
	48:4	816.5	0.11 ± 0.03	0.15 ± 0.09	0.1	1.39 ± 0.92
	48:3	818.5	1.50 ± 0.32	3.17 ± 0.73	0.8	**2.11 ± 0.67****
	48:2	820.5	2.78 ± 1.56	4.03 ± 1.84	1.5	1.45 ± 1.05
	48:1	822.5	4.95 ± 1.50	8.10 ± 4.50	<u>2.7</u>	1.64 ± 1.04
	48:0	824.5	3.93 ± 2.35	10.08 ± 8.06	2.1	**2.56 ± 2.55***
	50:5	842.5	0.18 ± 0.10	0.27 ± 0.10	0.1	1.53 ± 1.06
	50:4	844.5	0.67 ± 0.20	0.78 ± 0.28	0.4	1.15 ± 0.54
	50:3	846.5	10.07 ± 5.11	12.02 ± 5.94	<u>5.4</u>	1.19 ± 0.85
	50:2	848.5	11.31 ± 2.66	28.22 ± 7.01	<u>6.1</u>	**2.50 ± 0.85****
	50:1	850.5	1.20 ± 0.70	2.09 ± 0.43	0.6	1.75 ± 1.07
	50:0	852.5	3.09 ± 1.08	4.86 ± 2.51	1.7	1.57 ± 0.98
	52:6	868.5	0.28 ± 0.18	0.90 ± 0.49	0.1	**3.23 ± 2.76****
	52:5	870.5	5.15 ± 0.93	12.18 ± 4.96	<u>2.8</u>	**2.37 ± 1.05****
	52:4	872.5	7.41 ± 0.35	20.65 ± 8.27	<u>4.0</u>	**2.79 ± 1.12****
	52:3	874.5	48.81 ± 13.30	59.75 ± 20.62	<u>26.2</u>	1.22 ± 0.54
	52:2	876.5	3.59 ± 0.43	5.21 ± 2.40	1.9	1.45 ± 0.69
	52:1	878.5	3.99 ± 1.36	5.12 ± 2.12	2.1	1.28 ± 0.69
	52:0	880.5	0.29 ± 0.11	1.12 ± 0.60	0.2	**3.84 ± 2.49**
	54:7	894.5	0.65 ± 0.26	2.59 ± 1.48	0.4	**3.97 ± 2.78****
	54:6	896.5	5.50 ± 2.07	11.73 ± 5.21	<u>3.0</u>	**2.13 ± 1.24****
	54:5	898.5	11.98 ± 4.16	32.61 ± 15.39	<u>6.4</u>	**2.72 ± 1.59****
	54:4	900.5	18.56 ± 7.69	34.15 ± 8.54	<u>10.0</u>	1.84 ± 0.89**
	54:3	902.5	23.18 ± 13.45	33.10 ± 10.91	<u>12.5</u>	1.43 ± 0.95
	54:2	904.5	5.23 ± 1.40	8.67 ± 0.66	<u>2.8</u>	1.66 ± 0.46
	54:1	906.5	0.41 ± 0.19	0.79 ± 0.18	0.2	1.95 ± 1.02**
	54:0	908.5	0.12 ± 0.05	0.26 ± 0.20	0.1	**2.23 ± 1.98**
	56:8	920.5	0.40 ± 0.13	1.15 ± 0.48	0.2	**2.87 ± 1.54****
	56:7	922.5	0.53 ± 0.19	0.93 ± 0.29	0.3	1.73 ± 0.82**
	56:6	924.5	0.67 ± 0.15	1.44 ± 0.30	0.4	**2.17 ± 0.66****
	56:5	926.5	0.50 ± 0.12	0.99 ± 0.35	0.3	1.96 ± 0.83**
CE	16:1	640.5	4.54 ± 1.45	3.28 ± 0.43	1.1	0.72 ± 0.25*
(9)	16:0	642.5	3.21 ± 0.30	1.96 ± 0.67	0.8	0.61 ± 0.22**
	18:3	664.5	18.65 ± 2.28	9.09 ± 0.74	4.5	**0.49 ± 0.07****
	18:2	666.5	250.34 ± 84.49	118.15 ± 40.00	<u>60.5</u>	**0.47 ± 0.23****

	18:1	668.5	55.74 ± 15.51	28.44 ± 8.21	13.5	0.51 ± 0.20**
	20:5	688.5	6.92 ± 3.13	3.45 ± 0.59	1.7	**0.50 ± 0.24***
	20:4	690.5	49.64 ± 15.05	27.52 ± 8.07	12.0	0.55 ± 0.23**
	20:3	692.5	9.63 ± 2.31	5.05 ± 1.79	2.3	0.52 ± 0.22**
	22:6	714.5	15.13 ± 2.82	6.01 ± 1.91	3.7	**0.40 ± 0.15****

* $p < 0.05$; ** $p < 0.01$.

Table S2. Isomeric structure of PC, PE, and TG identified from CID spectra.

Saliva							
Class	Molecular species	Acyl chains	*m/z*	Class	Molecular species	Acyl chains	*m/z*
PC	32:1	16:0/16:1	732.554		48:3	16:1_16:1_16:1	818.723
		14:0/18:1	732.554			18:1_16:1_14:1	818.723
	34:1	16:0/18:1	760.585			18:2_16:0_14:1	818.723
		18:0/16:1	760.585			18:2_16:1_14:0	818.723
	36:3	18:1/18:2	784.585			18:2_18:1_12:0	818.723
		20:3/16:0	784.585		48:2	16:1_16:1_16:0	820.739
	36:2	18:0/18:2	786.601			18:0_16:1_14:1	820.739
		18:1/18:1	786.601			18:1_16:0_14:1	820.739
	38:4	18:0/20:4	810.601			18:1_16:1_14:0	820.739
		16:0/22:4	810.601			18:1_18:1_12:0	820.739
	38:2	20:0/18:2	814.632			18:2_16:0_14:0	820.739
		16:0/22:2	814.632			18:2_18:0_12:0	820.739
		18:0/20:2	814.632		48:1	16:1_16:0_16:0	822.755
	42:0	24:0/18:0	874.726			18:0_16:1_14:0	822.755
		26:0/16:0	874.726			18:1_16:0_14:0	822.755
	44:1	24:0/18:1	900.742		48:0	16:0_16:0_16:0	824.770
		18:0/24:1	900.742			18:0_16:0_14:0	824.770
PE	30:1	14:0/16:1	662.476		50:3	18:1_16:1_16:1	846.755
		16:0/14:1	662.476			18:2_16:1_16:0	846.755
		18:1/12:0	662.476			18:2_18:1_14:0	846.755
	30:0	16:0/14:0	664.491		50:2	18:0_16:1_16:1	848.770
		18:0/12:0	664.491			18:1_16:1_16:0	848.770
	32:2	16:1/16:1	688.491			18:1_18:1_14:0	848.770
		14:0/18:2	688.491			18:2_16:0_16:0	848.770
		18:1/14:1	688.491			18:2_18:0_14:0	848.770
	32:1	16:0/16:1	690.507		50:1	18:1_16:0_16:0	850.786
		14:0/18:1	690.507			18:1_18:0_14:0	850.786
	34:3	16:1/18:2	714.507		50:0	18:0_16:0_16:0	852.802
		16:0/18:3	714.507			18:0_18:0_14:0	852.802
		20:3/14:0	714.507		52:6	18:3_18:2_16:1	868.739
	34:2	16:0/18:2	716.523			18:3_18:3_16:0	868.739
		16:1/18:1	716.523		52:5	18:2_18:2_16:1	870.755
	34:1	16:0/18:1	718.538			18:3_18:1_16:1	870.755
		18:0/16:1	718.538			18:3_18:2_16:0	870.755
	36:3	18:1/18:2	742.538		52:4	18:2_18:1_16:1	872.770

		16:0/20:3	742.538
		18:3/18:0	742.538
	36:2	18:0/18:2	744.554
		18:1/18:1	744.554
		16:0/20:2	744.554
	36:1	18:0/18:1	746.569
		20:1/16:0	746.569
	38:5	16:0/22:5	766.538
		18:1/20:4	766.538
		20:3/18:2	766.538
	38:2	20:0/18:2	772.585
		20:1/18:1	772.585
		18:0/20:2	772.585
		22:2/16:0	772.585
TG	40:0	14:0_14:0_12:0	712.645
		16:0_12:0_12:0	712.645
	42:1	14:1_14:0_14:0	738.661
		16:0_14:1_12:0	738.661
		16:1_14:0_12:0	738.661
		18:1_12:0_12:0	738.661
	42:0	14:0_14:0_14:0	740.676
		16:0_14:0_12:0	740.676
		18:0_12:0_12:0	740.676
	44:3	16:1_14:1_14:1	762.661
		18:2_14:1_12:0	762.661
	44:2	16:0_14:1_14:1	764.676
		18:1_14:1_12:0	764.676
		18:2_14:0_12:0	764.676
	44:1	16:0_14:1_14:0	766.692
		16:1_14:0_14:0	766.692
		18:0_14:1_12:0	766.692
		18:1_14:0_12:0	766.692
	44:0	16:0_14:0_14:0	768.708
		16:0_16:0_12:0	768.708
		18:0_14:0_12:0	768.708
	46:3	16:1_16:1_14:1	790.692
		18:1_14:1_14:1	790.692
		18:2_14:1_14:0	790.692
		18:2_16:1_12:0	790.692
	46:2	16:1_16:0_14:1	792.708
		16:1_16:1_14:0	792.708
		18:1_14:1_14:0	792.708
		18:1_16:1_12:0	792.708
		18:2_14:0_14:0	792.708
		18:2_16:0_12:0	792.708
		18:2_18:2_16:0	872.770
	52:3	18:1_18:1_16:1	874.786
		18:2_18:0_16:1	874.786
		18:2_18:1_16:0	874.786
	52:2	18:1_18:0_16:1	876.802
		18:1_18:1_16:0	876.802
		18:2_18:0_16:0	876.802
	52:1	18:1_18:0_16:0	878.817
		18:0_18:0_16:0	880.833
	54:7	18:3_18:2_18:2	894.755
		18:3_18:3_18:1	894.755
		20:4_18:2_16:1	894.755
	54:6	18:2_18:2_18:2	896.770
		18:3_18:2_18:1	896.770
	54:5	18:2_18:2_18:1	898.786
		18:3_18:1_18:1	898.786
	54:4	18:2_18:1_18:1	900.802
		18:2_18:2_18:0	900.802
		18:3_18:1_18:0	900.802
		20:3_18:1_16:0	900.802
	54:3	18:1_18:1_18:1	902.817
		18:2_18:1_18:0	902.817
	54:0	18:1_18:0_18:0	906.849
		20:0_18:0_16:0	906.849
	56:8	20:4_18:2_18:2	920.770
		20:4_20:4_16:0	920.770
	56:7	20:3_18:2_18:2	922.786
		20:3_18:3_18:1	922.786
		20:3_20:3_16:1	922.786
		20:4_18:2_18:1	922.786
	56:5	20:1_18:3_18:1	926.817
		20:2_18:2_18:1	926.817
		20:2_18:3_18:0	926.817
		20:3_18:1_18:1	926.817
		20:3_18:2_18:0	926.817
		20:3_20:2_16:0	926.817
	56:4	20:0_18:2_18:2	928.833
		20:1_18:2_18:1	928.833
		20:2_18:1_18:1	928.833
	56:3	20:0_18:2_18:1	930.849
		20:1_18:1_18:1	930.849
	56:2	20:0_18:1_18:1	932.864
		20:1_18:1_18:0	932.864
		20:1_20:0_16:1	932.864
		20:1_20:1_16:0	932.864

	46:1	16:1_16:0_14:0	794.723			22:0_18:1_16:1	932.864
		18:0_16:1_12:0	794.723			22:0_18:2_16:0	932.864
		18:1_14:0_14:0	794.723			24:0_16:1_16:1	932.864
		18:1_16:0_12:0	794.723		58:8	20:4_20:4_18:0	948.801
	46:0	16:0_16:0_14:0	796.739			22:5_18:2_18:1	948.801
		18:0_14:0_14:0	796.739			22:6_18:1_18:1	948.801
		18:0_16:0_12:0	796.739				

Plasma

Class	Molecular species	Acyl chains	*m/z*	Class	Molecular species	Acyl chains	*m/z*
PC	32:2	14:0/18:2	730.538			18:2_14:1_14:0	790.692
		16:1/16:1	730.538			18:2_16:1_12:0	790.692
	32:1	14:0/18:1	732.554		46:2	16:1_16:0_14:1	792.708
		16:0/16:1	732.554			16:1_16:1_14:0	792.708
	34:4	14:0/20:4	754.538			18:1_14:1_14:0	792.708
		16:2/18:2	754.538			18:1_16:1_12:0	792.708
	34:3	14:0/20:3	756.554			18:2_14:0_14:0	792.708
		16:0/18:3	756.554			18:2_16:0_12:0	792.708
		16:1/18:2	756.554		46:1	16:0_16:0_14:1	794.723
	36:4	16:0/20:4	782.569			16:1_16:0_14:0	794.723
		18:2/18:2	782.569			18:1_14:0_14:0	794.723
	36:3	16:0/20:3	784.585			18:1_16:0_12:0	794.723
		18:1/18:2	784.585		48:4	18:2_16:1_14:1	816.708
	36:2	16:0/20:2	786.601			18:2_18:2_12:0	816.708
		18:0/18:2	786.601			18:3_18:1_12:0	816.708
		18:1/18:1	786.601		48:3	16:1_16:1_16:1	818.723
	36:1	16:0/20:1	788.616			18:1_16:1_14:1	818.723
		18:0/18:1	788.616			18:2_16:0_14:1	818.723
	38:7	16:1/22:6	804.554			18:2_16:1_14:0	818.723
		18:2/20:5	804.554			18:2_18:1_12:0	818.723
	38:6	16:0/22:6	806.569		48:2	16:1_16:1_16:0	820.739
		18:1/20:5	806.569			18:1_16:0_14:1	820.739
		18:2/20:4	806.569			18:1_16:1_14:0	820.739
	38:5	16:0/22:5	808.585			18:1_18:1_12:0	820.739
		18:0/20:5	808.585			18:2_16:0_14:0	820.739
		18:1/20:4	808.585		48:1	16:1_16:0_16:0	822.755
	38:4	16:0/22:4	810.601			18:0_16:1_14:0	822.755
		18:0/20:4	810.601			18:1_16:0_14:0	822.755
		18:1/20:3	810.601		48:0	16:0_16:0_16:0	824.770
	38:2	18:0/20:2	814.632			18:0_16:0_14:0	824.770
		20:0/18:2	814.632		50:5	18:2_18:2_14:1	842.723
		20:1/18:1	814.632			18:3_16:1_16:1	842.723
	40:8	20:4/20:4	830.569			18:3_18:2_14:0	842.723
		22:6/18:2	830.569		50:4	18:1_16:2_16:1	844.739
	40:7	18:1/22:6	832.585			18:2_16:1_16:1	844.739

		20:3/20:4	832.585		18:2_18:1_14:1	844.739
		22:5/18:2	832.585		18:2_18:2_14:0	844.739
	40:6	18:0/22:6	834.601		18:3_16:1_16:0	844.739
		18:1/22:5	834.601		18:3_18:1_14:0	844.739
		20:2/20:4	834.601	50:3	18:1_16:1_16:1	846.755
		20:3/20:3	834.601		18:1_18:1_14:1	846.755
PE	36:5	16:0/20:5	738.507		18:2_18:1_14:0	846.755
		16:1/20:4	738.507	50:2	18:1_16:1_16:0	848.770
	36:4	16:0/20:4	740.522		18:1_18:1_14:0	848.770
		18:2/18:2	740.522		18:2_16:0_16:0	848.770
		18:1/18:3	740.523	50:1	18:0_16:1_16:0	850.786
	36:3	16:0/20:3	742.538		18:1_16:0_16:0	850.786
		18:1/18:2	742.538	52:6	18:2_18:2_16:2	868.739
	36:1	16:0/20:1	746.569		18:3_18:2_16:1	868.739
		18:0/18:1	746.569		20:4_16:1_16:1	868.739
	38:6	18:2/20:4	764.522	52:5	18:2_18:2_16:1	870.755
		22:6/16:0	764.522		18:3_18:1_16:1	870.755
		18:1/20:5	764.523		18:3_18:2_16:0	870.755
	38:5	16:0/22:5	766.538	52:4	18:2_18:1_16:1	872.770
		18:0/20:5	766.538		18:2_18:2_16:0	872.770
		18:1/20:4	766.538		18:3_18:1_16:0	872.770
	38:4	18:0/20:4	768.554	52:3	18:1_18:1_16:1	874.786
		18:1/20:3	768.554		18:2_18:1_16:0	874.786
	40:6	18:0/22:6	792.554	52:2	18:1_18:0_16:1	876.802
		18:1/22:5	792.554		18:1_18:1_16:0	876.802
	40:5	18:0/22:5	794.569		18:2_18:0:16:0	876.802
		20:1/20:4	794.569	52:1	18:0_18:0_16:1	878.817
TG	40:1	14:1_14:0_12:0	710.629		18:1_18:0_16:0	878.817
		16:1_12:0_12:0	710.629	54:7	18:3_18:2_18:2	894.755
	40:0	14:0_14:0_12:0	712.645		20:4_18:2_16:1	894.755
		16:0_12:0_12:0	712.645	54:6	18:2_18:2_18:2	896.770
	42:2	16:1_14:1_12:0	736.645		18:3_18:2_18:1	896.770
		16:1_14:1_12:0	736.645		20:4_18:1_16:1	896.770
		18:2_12:0_12:0	736.645	54:5	18:2_18:2_18:1	898.786
	42:1	14:1_14:0_14:0	738.661		18:3_18:1_18:1	898.786
		16:0_14:1_12:0	738.661		20:3_18:2_16:0	898.786
		16:1_14:0_12:0	738.661	54:4	18:2_18:1_18:1	900.802
		18:1_12:0_12:0	738.661		18:2_18:2_18:0	900.802
	42:0	14:0_14:0_14:0	740.676		20:3_18:1_16:0	900.802
		16:0_14:0_12:0	740.676	54:3	18:1_18:1_18:1	902.817
	44:2	16:0_14:1_14:1	764.676		18:2_18:1_18:0	902.817
		16:1_14:1_14:0	764.676		20:2_18:1_16:0	902.817
		16:1_16:1_12:0	764.676	54:2	18:1_18:1_18:0	904.833
		18:1_14:1_12:0	764.676		20:1_18:1_16:0	904.833
		18:2_14:0_12:0	764.676	54:0	18:0_18:0_18:0	908.864

44:1	16:0_14:1_14:0	766.692		24:0_16:0_14:0	908.864
	16:1_14:0_14:0	766.692	56:8	20:4_18:2_18:2	920.770
	16:1_16:0_12:0	766.692		22:6_18:2_16:0	920.770
	18:1_14:0_12:0	766.692	56:7	20:4_18:2_18:1	922.786
44:0	16:0_14:0_14:0	768.708		20:5_18:1_18:1	922.786
	16:0_16:0_12:0	768.708		22:5_18:2_16:0	922.786
	18:0_14:0_12:0	768.708	56:6	20:4_18:1_18:1	924.802
46:3	16:1_16:1_14:1	790.692		22:4_18:2_16:0	924.802
	18:1_14:1_14:1	790.692		22:5_18:1_16:0	924.802

Table S3: List of lipid standards used for this study. Lipids marked with * were used to prepare an internal standard lipid mixture.

No.	Class	Acyl Chains	No.	Class	Acyl Chains
1	LPC	16:0	25	LPG	17:1
2	LPC	17:1	26	LPG	13:0*
3	LPC	18:1-D_7*	27	PG	17:0/17:0
4	PC	15:0/18:1-D_7*	28	PG	15:0/18:1-D_7*
5	PC	16:0/16:0	29	LPI	17:1
6	PC	17:0/17:0	30	LPI	13:0*
7	PC	18:1/18:0	31	PI	16:0-D_{31}/18:1
8	EtherPC	P-18:0/18:1-D_9	32	PI	15:0/18:1-D_7*
9	LPE	14:0	33	SM	d18:1/17:0
10	LPE	17:1	34	SM	d18:1/18:1-D_9*
11	LPE	18:1-D_7*	35	Cer	d18:1/17:0
12	PE	12:0/12:0	36	Cer	d18:1-D_7/24:1
13	PE	16:0/16:0	37	Cer	d18:1-D_7/24:0*
14	PE	15:0/18:1-D_7*	38	HexCer	d18:1/17:0
15	PE	17:0/17:0	39	HexCer	d18:1-D_7/15:0*
16	EtherPE	P-18:0/18:1-D_9	40	Hex2Cer	d18:1/16:0
17	LPA	17:0*	41	Hex2Cer	d18:1/17:0
18	LPA	17:1	42	Hex2Cer	d18:1-D_7/15:0*
19	PA	15:0/18:1-D_7*	43	DG	15:0/18:1-D_7*
20	PA	17:0/17:0	44	DG	1,3-18:0-D_5
21	LPS	17:1	45	TG	15:0/18:1-D_7/15:0*
22	LPS	13:0*	46	TG	17:0/17:1/17:0-D_5
23	PS	17:0/17:0	47	CE	17:0
24	PS	15:0/18:1-D_7*	48	CE	18:1-D_7*

Table S4: Type of precursor and quantifier ions of each lipid class and collision energy for SRM quantification

No.	Class	Acyl chain calibration standard	Acyl chain Internal standard	Type of precursor /quantifier ions	m/z calibration standard precursor ion	m/z calibration standard quantifier ion	m/z internal standard precursor ion	m/z internal standard quantifier ion	Collision energy (V)
1	LPC	17:01	18:1-D_7	$[M+H]^+/[Pcho+H]^+$	508.5	184.5	529.5	184.5	40
2	PC	17:0/17:0	15:0/18:1-D_7	$[M+H]^+/[Pcho+H]^+$	762.5	184.5	753.5	184.5	40
3	Ether PC	P-18:0/18:1-D_9	PC 15:0/18:1-D_7	$[M+H]^+/[Pcho+H]^+$	781.5	184.5	753.5	184.5	40
4	LPE	17:01	18:1-D_7	$[M+H]^+/[M+H-141]^+$	466.5	325.5	487.5	346.5	20
5	PE	17:0/17:0	15:0/18:1-D_7	$[M+H]^+/[M+H-141]^+$	720.5	579.5	711.5	570.5	20
6	Ether PE	P-18:0/18:1-D_9	PE 15:0/18:1-D_7	$[M+H]^+/[M+H-RCOOCH_2CHCH_2OH]^+$	739.5	512.5	711.5	570.5	30
7	LPA	17:00	17:01	$[M-H]^-/[M-H-RCOOH]^-$	423.5	269.5	421.5	267.5	35
8	LPG	17:01	13:00	$[M-H]^-/[RCOO]^-$	495.5	267.5	441.5	213.5	35
9	PG	17:0/17:0	15:0/18:1-D_7	[M-H]-/[RCOO]-	749.5	269.5	740.5	241.5	35
10	LPI	17:01	13:00	$[M-H]^-/[RCOO]^-$	583.5	267.5	529.5	213.5	35
11	PI	16:0-D_{31}/18:1	15:0/18:1-D_7	$[M-H]^-/[RCOO]^-$	865.5	286.5	828.5	241.5	35
12	SM	d18:1/17:0	d18:1/18:1-D_9	$[M+H]^+/[Pcho+H]^+$	717.5	184.5	738.5	184.5	40
13	Cer	d18:1-D_7/24:1	d18:1-D_7/24:0	$[M+H]+/[d18:1]^+$	655.5	271.5	657.5	271.5	30
14	Hex Cer	d18:1/17:0	d18:1-D_7/15:0	$[M+H]^+/[d18:1]^+$	714.5	271.5	693.5	264.5	30
15	Hex2 Cer	d18:1/17:0	d18:1-D_7/13:0	$[M+H]^+/[d18:1]^+$	876.5	271.5	855.5	264.5	50
16	DG	1,3-18:0-D_5	15:0/18:1-D_7	$[M+NH_4]^+/[M+H-RCOOH]^+$	647.5	341.5	605.5	290.5	35
17	TG	17:0/17:1/17:0-D_5	15:0/18:1-D_7/15:0	$[M+NH_4]^+/[M+H-RCOOH]^+$	869.5	582.5	829.5	514.5	35
18	CE	17:00	18:1-D_7	$[M+NH_4]^+/[Chole]^+$	656.5	369.5	675.5	369.5	30

※ 141 Da : ethanolamine, Pcho : phosphocholine, Chole : Cholesterol

Table S5: Slopes and intercepts of the calibration curve of each lipid class. Numbers with red color represent the negative value.

Saliva			
class	slope	y-intercept	R^2
LPC	1.9913	0.0585	0.998
PC	4.2368	(0.1765)	0.997
EtherPC	1.2347	0.1388	0.992
LPE	1.9064	0.0464	0.993
PE	14.2269	(0.3364)	0.992
EtherPE	1.8008	0.2004	0.992
LPA	2.0119	0.0064	0.994
PA	0.4716	0.0057	0.993
LPS	0.9314	0.3693	0.995
PS	0.8169	0.2198	0.995
LPG	7.7986	(0.0219)	0.995
PG	1.9112	(0.0670)	0.996
LPI	0.0792	0.0192	0.999
PI	0.7823	0.0037	0.996
SM	0.8927	0.8497	0.994
Cer	4.2431	0.1077	0.999
HexCer	2.3422	0.0072	0.990
Hex2Cer	0.2956	0.0586	0.996
DG	0.4466	0.0054	1.000
TG	0.7076	0.0323	0.999
CE	0.5914	0.0630	0.997
Plasma			
class	slope	y-intercept	R^2
LPC	2.6673	0.2034	0.997
PC	4.4555	(0.0463)	0.995
EtherPC	2.2140	(0.0049)	0.999
LPE	2.2037	(0.0832)	0.996
PE	0.9412	0.0139	0.988
EtherPE	0.9986	(0.0192)	0.984
LPG	0.1864	(0.0106)	0.994
PG	0.5927	(0.0356)	0.992
LPI	0.1218	(0.0012)	0.991
PI	0.4714	0.0371	0.997
SM	1.2610	0.0211	0.989
Cer	1.5294	(0.0330)	0.996
HexCer	1.1734	0.0299	0.993
DG	1.5608	0.1109	0.996
TG	0.8722	(0.0344)	0.997

CE	2.4883	(0.0957)	0.984

Table S6. Limit of detection (LOD) and limit of quantitation (LOQ) values of external lipid standards based on calibration curves and the type of internal standards spiked to lipid extracts by nUHPLC-ESI-MS/MS.

Saliva				
class	external standard	internal standard	LOD (pmol)	LOQ (pmol)
LPC	17:1	18:1-D_7	0.05	0.17
PC	17:0/17:0	15:0/18:1-D_7	0.09	0.29
EtherPC	P-18:0/18:1-D_9	PC 15:0/18:1-D_7	0.07	0.24
LPE	17:1	18:1-D_7	0.04	0.12
PE	17:0/17:0	15:0/18:1-D_7	0.13	0.42
EtherPE	P-18:0/18:1-D_9	PE 15:0/18:1-D_7	0.08	0.26
LPA	17:0	17:1	0.02	0.06
PA	17:0/17:0	15:0/18:1-D_7	0.08	0.27
LPS	17:1	13:0	0.21	0.71
PS	17:0/17:0	15:0/18:1-D_7	0.10	0.35
LPG	17:1	13:0	0.06	0.20
PG	17:0/17:0	15:0/18:1-D_7	0.11	0.36
LPI	17:1	13:0	0.09	0.30
PI	16:0-D_{31}/18:1	15:0/18:1-D_7	0.00	0.01
SM	d18:1/17:0	d18:1/18:1-D_9	0.12	0.40
Cer	d18:1-D_7/24:1	d18:1-D_7/24:0	0.03	0.10
HexCer	d18:1/17:0	d18:1-D_7/15:0	0.07	0.25
Hex2Cer	d18:1/17:0	d18:1-D_7/15:0	0.01	0.03
DG	1,3-18:0-D_5	15:0/18:1-D_7	0.01	0.04
TG	17:0/17:1/17:0-D_5	15:0/18:1-D_7/15:0	0.02	0.05
CE	17:0	18:1-D_7	0.07	0.25
Plasma				
class	external standard	internal standard	LOD (pmol)	LOQ (pmol)
LPC	17:1	18:1-D_7	0.09	0.29
PC	17:0/17:0	15:0/18:1-D_7	0.04	0.14
EtherPC	P-18:0/18:1-D_9	PC 15:0/18:1-D_7	0.06	0.21
LPE	17:1	18:1-D_7	0.06	0.19
PE	17:0/17:0	15:0/18:1-D_7	0.05	0.18
EtherPE	P-18:0/18:1-D_9	PE 15:0/18:1-D_7	0.04	0.14
LPG	17:1	13:0	0.02	0.06
PG	17:0/17:0	15:0/18:1-D_7	0.10	0.34
LPI	17:1	13:0	0.08	0.27
PI	16:0-D_{31}/18:1	15:0/18:1-D_7	0.08	0.25
SM	d18:1/17:0	d18:1/18:1-D_9	0.13	0.43

Cer	d18:1-D_7/24:1	d18:1-D_7/24:0	0.05	0.18
HexCer	d18:1/17:0	d18:1-D_7/15:0	0.05	0.18
DG	1,3-18:0-D_5	15:0/18:1-D_7	0.10	0.33
TG	17:0/17:1/17:0-D_5	15:0/18:1-D_7/15:0	0.03	0.08
CE	17:0	18:1-D_7	0.07	0.25

※ Limit of detection = 3 × standard deviation of y-intercept / slope,
Limit of qunatitation = 10 × standard deviation of y-intercept / slope

Table S7. Characteristics of patients with lung cancer and healthy controls.

**Examined Parameter**	**LC Patients (%) (n=26)**	**Controls (%) (n=30)**
**Age (range)**	62.1 (44–82)	59.5 (41–80)
**Gender**		
Female	11 (42.3)	13 (43.3)
Male	15 (57,7)	17 (56.7)
**Smoking status**		
Never	4 (15.4)	7 (23.3)
Previous	19 (73.1)	16 (53.3)
Current	3 (11.5)	7 (23.3)
**Comorbid diseases**		
Cardiovascular disease	2 (7.7)	2 (6.7)
Hypertension	7 (26.9)	8 (26,7)
Diabetes mellitus	2 (7.7)	3 (10)
**Staging according to TNM**		
I	6 (23.1)	
II	3 (11.5)	
III	10 (38.5)	
IV	7 (26.9)	
**Pathological classification**		
Adenocarcinoma	19 (73.1)	
Squamous carcinoma	7 (26.9)	
**Tumor location**		
left-side	9 (34.6)	
right-side	17 (65.4)	
**Anatomical type**		
Peripheral carcinoma	21 (80.8)	
Central carcinoma	5 (19.2)	
**Treatment**		

**Examined Parameter**	**LC Patients (%) (n=26)**	**Controls (%) (n=30)**
Chemotherapy	19 (73.1)	
Chemotherapy+Immunotherapy	4 (15.4)	
Chemotherapy+Radiotherapy	3 (11.5)	
